# Advancements in thermochemical predictions: a multi-output thermodynamics-informed neural network approach

**DOI:** 10.1186/s13321-025-01033-0

**Published:** 2025-06-16

**Authors:** Raheel Hammad, Sownyak Mondal

**Affiliations:** Tata Institute of Fundamental Research Hyderabad, Hyderabad, Telangana 500046 India

**Keywords:** American Chemical Society

## Abstract

The Gibbs free energy of an inorganic material represents its maximum reversible work potential under constant temperature and pressure. Its calculation is crucial for understanding material stability, phase transitions, and chemical reactions, thus guiding optimization for diverse applications like catalysis and energy storage. In this study, we have developed a Physics-Informed Neural Network model that leverages the Gibbs free energy equation. The overall loss function is adjusted to allow the model to simultaneously predict all three thermodynamic quantities, including Gibbs free energy, total energy, and entropy, thus transforming it into a multi-output model. In recent literature, there is a growing emphasis on evaluating machine learning models under challenging conditions, such as small datasets and out-of-distribution predictions. Reflecting this trend, we have rigorously benchmarked our model across these scenarios, demonstrating its robustness and adaptability. It turns out that our model demonstrates a 43% improvement for normal scenario and even more in out-of-distribution regime compared to the next-best model.

**Scientific Contribution** This study introduces the application of a Physics-Informed Neural Network to simultaneously compute multiple thermodynamic properties, including Gibbs free energy, total energy, and entropy. By integrating the Gibbs free energy equation into the loss function, the model achieves superior accuracy in low data regimes and enhances robustness in the out-of-distribution scenarios.

## Introduction

In material science, accurately predicting the thermochemical properties is of utmost importance. Accurate calculation of the Gibbs free energy enables researchers to understand and predict thermodynamic stability, phase transitions, and chemical reactions in materials.[1–3] Experimentally, It can be determined through methods such as calorimetry, electrochemical measurements, and phase equilibrium experiments from thermochemical properties like heat and energy of formation.[4, 5] However, these experimental methods can be costly, tedious, and sometimes limited by the availability of samples.

As a result, considerable research effort has been directed towards utilizing computer simulations to determine these thermodynamic properties. In conjunction with thermodynamic integration, molecular dynamics and Monte Carlo techniques have been employed to calculate the free energy for various systems, like ionic hydration and biomolecules.[6–10] Density Functional Theory (DFT) also provides a framework to predict the thermodynamic properties of materials by considering the electronic structure and interactions within a system.[11, 12] Density Functional Perturbation Theory (DFPT) is an extension of DFT that allows for the calculation of vibrational properties and phonon modes, which are essential for determining the Gibbs free energy at finite temperatures.[13] Togo et al. introduced an open-source code called Phonopy, which can handle force constants obtained from DFPT and calculate various properties of crystalline materials, such as entropy, Gibbs free energy, and specific heat etc.[14, 15] There are studies, where ab initio Molecular Dynamics has been utilized to estimate free energy.[16] Even though the computational techniques mentioned above provide valuable insight into the estimation of thermochemical properties, they often require significant computational resources and can be time-consuming.

In recent years, the substantial data generated through computational and experimental methods, along with the application of advanced machine learning (ML) techniques, has propelled the field of materials science into a new era often described as the fourth paradigm of scientific exploration [17]. Different ML algorithms are utilized to generate efficient and accurate predictions of mechanical and electronic properties of materials [18–21]. Using solely chemical descriptors, Legrian et al. constructed an ML model capable of predicting thermodynamic properties, such as vibrational entropies and free energies, of crystalline compounds [22]. Bartel et al. utilized the SISSO approach to develop a set of simple and accurate descriptors for predicting the Gibbs energy of stoichiometric inorganic compounds with high resolution across a wide temperature range [23]. These descriptors enable the accurate prediction of reaction energetics for solid-state reactions, offering insights into materials stability, synthesizability, and the temperature-dependent scale of metastability for inorganic compounds. Laiu et al. proposed a data-centric deep learning approach using neural networks to predict the thermodynamic properties of ternary solid solutions [24].

In machine learning, the ability to construct models with minimal data is crucial for applications where data scarcity or high-dimensional spaces pose challenges [25]. Physics-informed neural networks (PINNs) offer a promising solution by incorporating domain-specific knowledge and physical principles into the model architecture [26–31]. By integrating physics-based constraints and relationships, these models can effectively leverage limited data to make accurate predictions and enhance the robustness of the machine learning framework [32]. Guan et al. introduced a framework for differentiable thermodynamic modeling, integrating phase equilibria and thermochemical quantities into the loss function, which represents a significant advancement in optimizing thermodynamic models using differentiable programming [33].

**Fig. 1:**
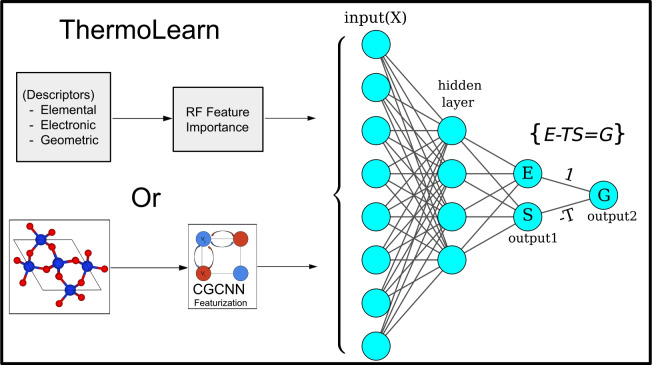
Schematic of ThermoLearn architecture. Featurization can be done using either CGCNN or elemental/crystal properties. Total Energy and Entropy are collected from output1, while Free energy is obtained from output2

In this study, we leveraged the Gibbs free energy equation which gives rise to a configuration that enables the model to predict all three thermodynamic properties: free energy, entropy, and total energy simultaneously. This model (Figure 1), termed ThermoLearn (Thermodynamics-learned Neural Network), demonstrates superiority over existing algorithms in different scienarios. The model is benchmarked on two distinctly different datasets-one experimental and the other computational. Notably, both datasets are significantly small (details provided in the Data Extraction section) This attribute is particularly vital in fields such as chemistry and materials science, where data often exist in limited quantities. Furthermore, ThermoLearn demonstrates an exceptional ability to assimilate domain-specific knowledge, which enhances its performance on unseen datasets. To illustrate this, we demonstrated that the model exhibits superior predictive accuracy in scenarios where the test set (out of distribution data) significantly deviates from the distribution characteristics of the training set. Additionally, it has been demonstrated that ThermoLearn performs effectively irrespective of the feature representation techniques employed, such as crystal-compositional features or graph-based features. This robustness is attributed to ThermoLearn’s ability to pick up underlying physical principles, enabling accurate predictions across different methods. Last but not least, we demonstrated the model’s accuracy by predicting the variation of all thermodynamic quantities as a function of temperature for several materials.

## Methods

### Data extraction and feautrization

The National Institute of Standards and Technology (NIST-JANAF) database [34], renowned for its meticulously curated experimental data, serves as a cornerstone in materials science research. This collection encompasses data regarding free energy, total energy, and entropy, specifically focusing on materials in the gas phase and at a temperature of 1200 K. We will refer to this experimental dataset as ‘JANAF’ for the rest of the paper. This dataset contains a total of 694 materials.

**Fig. 2:**
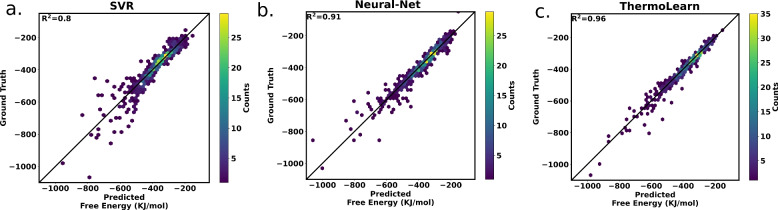
Performance comparison for the prediction of Gibbs Free Energy on NIST-JANAF dataset for **a** Support vector regression (SVR) and **b** neural network and **c** thermoLearn

In conjunction with the JANAF, we have also extracted a dataset from PhononDB [14, 15]. The PhononDB database offers invaluable insights into the phonon dispersion, density of states, and related thermal properties across a diverse range of materials. As our goal was to focus on small datasets, we specifically choose the metal oxide compounds, extracting pertinent data such as free energy, total energy, and entropy at varying temperatures. The dataset consists of a total of 873 materials. Throughout the paper, we will refer to this dataset as the ‘PhononDB’.

Featurization plays a pivotal role in machine learning tasks, serving as a bridge between raw data and model inputs. In our study utilizing the PhononDB dataset, we leverage both crystal and elemental features to characterize materials comprehensively. The crystal features like bond lengths, lattice parameters, etc. are collected from the Materials Project database [35] since the PhononDB dataset contains the material IDs as well. The elemental descriptors are quantified through mathematical expressions denoting their average, minimum, maximum, and difference values, respectively. Additionally, we have used graph-based features using CGCNN [36] for the PhononDb dataset.

However, the experimental JANAF dataset being devoid of the structural information is feature-engineered solely based on the compositions of the materials. Similar to the previous dataset, the same mathematical expressions have been employed here to characterize the elemental properties.

### ThermoLearn details

Since many of the previously mentioned features are redundant, a selection process is conducted using the Random Forest algorithm to optimize the feature space. Subsequently, the selected features are fed into a Feedforward Neural Network (FNN). The penultimate layers of this FNN are trained on the energy and entropy values of the Thermodynamic Free Energy equation:

$$\begin{aligned} G=E-TS. \end{aligned}$$

Here, ‘T’ represents the temperature of the system. Furthermore, to constrain the network to this equation we train it using a modified loss function described below. This loss function is formulated as a linear combination of three mean square error (MSE) terms, namely $MSE_U$, $MSE_S$, and $MSE_{Thermo}$.

1 $$\begin{aligned} L=w_1 \times MSE_{E}+w_2 \times MSE_{S}+w_3 \times MSE_{Thermo} \end{aligned}$$

where $MSE_{E}$ and $MSE_{S}$ are the regular loss functions for Energy and Entropy. While $MSE_{Thermo}$ is the thermodynamic loss defined as

2 $$\begin{aligned} MSE_{thermo}=MSE(E_{pred}-S_{pred} \times T,G_{obs} ) \end{aligned}$$

The weights ‘$w_i$’ in equation 1 serve as hyperparameters to balance the influence of each MSE term during training. These hyperparameters play a crucial role in adjusting the weights of individual MSE terms, considering their varying magnitudes and importance in the learning process. For each layer of the Feedforward Neural Network, we use the Leaky ReLU activation function, which effectively handles vanishing gradient problems[37]. The network is trained to minimize the loss (L) using ADAM, a stochastic gradient descent method [38]. In Eq. (2), $E_{pred}$ and $S_{pred}$ are the predicted values of energy and entropy respectively. Whereas $G_{obs}$ are the observed values of Free energy. All the network hyperparameters were optimized to maximize the coefficient of determination $R^2$ for the Free Energy. Details of the hyperparameter optimization are provided in the supplementary information. The codes are made available in https://github.com/Sudo-Raheel/ThermoLearn

**Table 1:** Training processes for different datasets

Dataset name	Feature type	Training process
NIST-JANAF	Elemental features	4-fold cross-validation
PhononDB	Elemental + Crystal	4-fold cross-validation
NIST-JANAF-OOD	Elemental	Train on Cluster-1, Test on Cluster-2
PhononDB-OOD	Elemental + Crystal	Train on Cluster-1, Test on Cluster-2

**Fig. 3:**
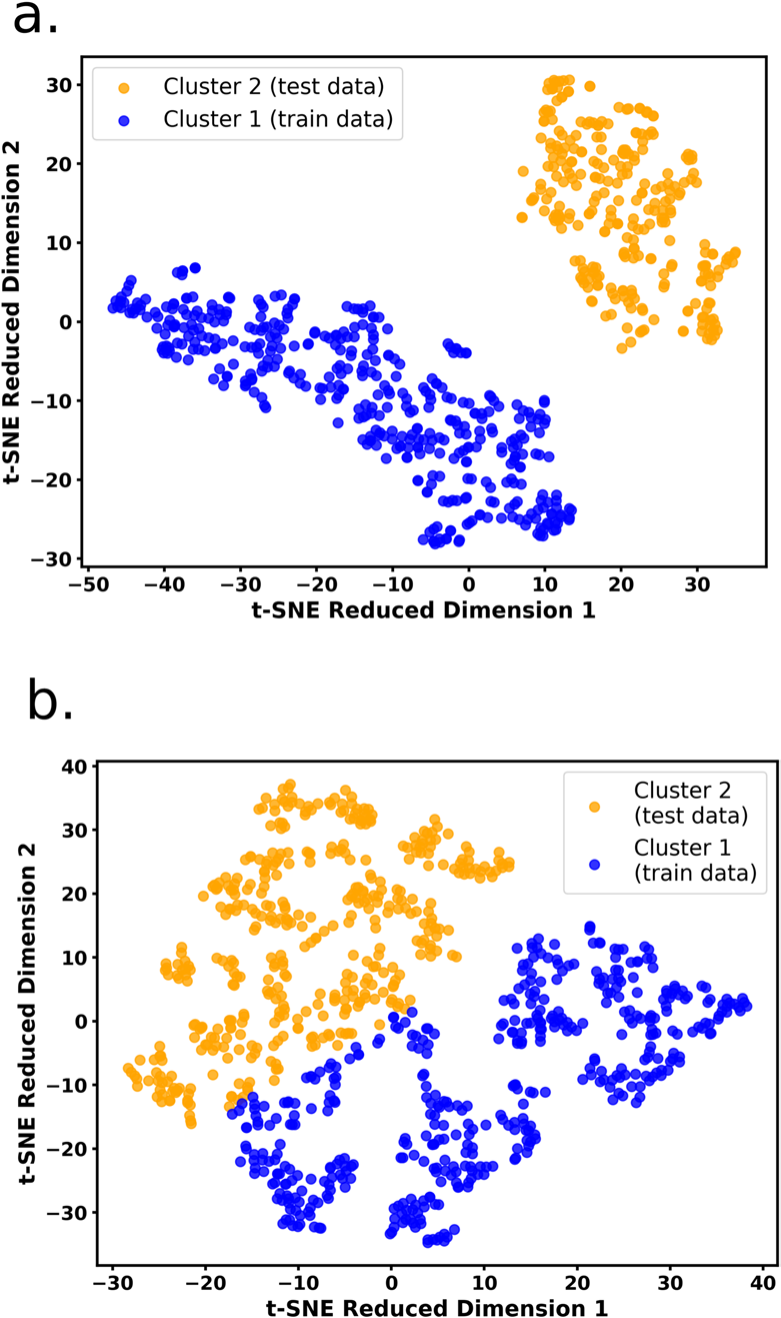
Formation of clusters (colour is based on K-means clustering) in **a** NIST-JANAF database and **b** PhononDB database

### Other ML models

In this study, we employed a suite of specialized machine learning models tailored for materials science, alongside more general algorithms. The specialized models included Crystal Graph Convolutional Neural Network (CGCNN) [36], MatErials Graph Network (MEGNet) [39], Material Optimal Descriptor Network (MODNet) [40], and Compositionally-Restricted Attention-Based Network (CrabNet) [41]. For broader applicability, we utilized ensemble methods such as Random Forest Regression, Gradient Boosting Regression, AdaBoost, and XGBoost. Additionally, we implemented several regression techniques including Ridge Regression, Support Vector Regression (SVR) and vanilla neural network to benchmark against our method.

Table 1 presents a summary of the various experiments conducted in this study, with further details provided in the Results and Discussion section.

## Results and discussion

**Table 2:** Combined comparison of RMSE values across JANAF dataset and Metal Oxide PhononDb using various machine learning methods

Dataset	JANAF						
Method	AdaBoost	Ridge	SVR	GBR	XGB	NN	ThermoLearn
RMSE (KJ/mol)	51.93	47.72	47.53	42.35	36.11	32.19	22.51
Dataset	Metal oxide PhononDb						
Method	MEGNet	GBR	CGCNN	XGB	Ridge	NN	ThermoLearn
RMSE (KJ/mol)	70.23	45.46	31.900	41.45	34.36	19.00	14.20

**Fig. 4:**
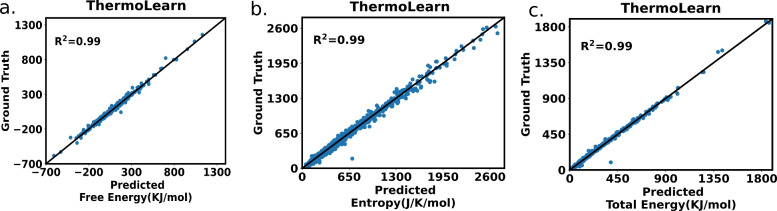
Ground truth versus ThermoLearn predicted values for all three thermodynamic quantities (PhononDB dataset). **a** Free energy, **b** entropy and **c** total energy

It is recognized that chemical datasets are usually smaller and more diverse compared to other fields. Although both the datasets used in this study, are for inorganic materials, they are very different from each other due to inherently different referencing used for collecting the properties. Moreover, the featurization of these datasets differs significantly from each other. Although these datasets may be bunched under the umbrella term of “Free energy” the truth is they are incompatible with each other, which means the hope of combining these datasets for direct use or transfer learning goes out of the window. To further complicate the issue, even within a single dataset, the presence of clustered data points can significantly impair the performance of machine learning models when applied to clusters that differ from those on which they were trained. This phenomenon, known as out-of-distribution (OOD) prediction, presents a considerable challenge. In this study, we intend to alleviate these issues of small datasets and robustness against OOD prediction by incorporating laws of physics in the model. One important thing about thermodynamic data is, that irrespective of the simulation method we usually compute the partition function of the system, using which we can access different thermodynamic quantities like Energy, Free Energy, Entropy, etc. One shortcoming of general regression methods is that these require quite a lot of data, since a single target quantity is used. Separate models need to be built to predict different target quantities. Constructing these regression models for each thermodynamic quantity can be labor-intensive due to the need for individualized featurization and algorithm selection. Moreover, building these models for small datasets presents challenges related to overfitting and poor performance in OOD regime. However, ThermoLearn offers several advantages over this approach. Firstly, it is trained on all three thermodynamic quantities (Free energy, total energy, and entropy) simultaneously, streamlining the modeling process. Secondly, the thermodynamic constraint allows us to handle small datasets effectively, hence mitigating the risk of overfitting and poor performance in the OOD regime commonly encountered in such scenarios. Finally, empirical evidence suggests that ThermoLearn exhibits superior performance in terms of accuracy and robustness compared to traditional regression models, making it a promising tool for thermodynamic property prediction. Although this study focuses on Gibbs Free Energy and collects quantities like entropy and total energy as a byproduct of the architecture, one can also build it the other way around.

### Benchmark

In the materials science community, the reliability of experimental datasets often surpasses that of computed data. Therefore, we chose the JANAF dataset to conduct an in-depth comparative analysis between existing regression algorithms and ThermoLearn. We use 4-fold cross-validation to compute the performance of the model on the full dataset (Fig. 2). These results are shown in Table 2. Algorithms such as AdaBoost, and Ridge have exhibited subpar performance in predictive modeling tasks. On the other hand, gradient boosting regression (GBR), random forest regression (RFR), extreme gradient boosting (XGB), and neural network regression algorithms have demonstrated comparatively favorable results. Notably, in all the algorithms, the hyperparameters are tuned to maximize the coefficient of determination ($R^2$) score. However, ThermoLearn demonstrates an outstanding $43\%$ reduction in Root Mean Square Error (RMSE) compared to the next best model, showcasing its significant enhancement in predictive accuracy. Additionally, we have benchmarked the performance of ThermoLearn for the PhonoDb dataset against the above-discussed algorithms. As seen with the JANAF dataset, ThermoLearn once again outperforms the rest as seen in Table 2.

### Out of distribution

Models trained on extensive datasets often exhibit artificially high-performance metrics when evaluated using conventional train-test splits or k-fold validation methods. Sadman et al. has shown a decrease in the performance of well-known models like CGCNN, MEGNET [39] while making predictions in the OOD regime. Given that our investigation focuses on smaller datasets, the susceptibility to inflated performance metrics in our models is likely even more pronounced.

**Table 3:** Combined comparison of RMSE and $R^2$ values across JANAF dataset and Metal Oxide PhononDb using various machine learning methods for OOD prediction

Dataset	JANAF		
Method	XGB	NN	ThermoLearn
RMSE (KJ/mol)	49.840	49.489	28.984
${R}^2$	0.799	0.814	0.936
Dataset	Metal oxide PhononDb		
Method	XGB	NN	ThermoLearn
RMSE (KJ/mol)	23.783	23.876	14.794
${R}^2$	0.824	0.822	0.931

#### OOD datasets

To reduce the dimensionality of the feature space while preserving the local structure of the data, t-distributed Stochastic Neighbour Embedding (t-SNE) was applied to both the original datasets (with top 25 features of JANAF and top 35 features for PhononDB). Following dimensionality reduction, the unsupervised K-Means clustering method was directly performed on the two-dimensional data obtained from the t-SNE transformation to categorize the data points into two distinct clusters. The experimental dataset consists of 419 data points in Cluster 1, which are used for training, while Cluster 2 contains the remaining 275 data points, which serve as the test set. Similarly, for the PhononDB dataset, Cluster 1 includes 452 points for training, and Cluster 2 comprises 421 data points for testing (Fig. 3).

#### OOD results

In Table 3, it is evident that ThermoLearn outperforms other algorithms. Nonetheless, other models such as XGBoost and conventional neural networks demonstrate respectable performance. Consequently, we extend our experimentation to include these high-performing models from Table 3, testing them under out-of-distribution (OOD) conditions. This is done by randomly selecting 80% of the data from both training and testing clusters. The chosen algorithms, as previously mentioned, are applied to train and test using this subset, having undergone hyperoptimization to maximize the $R^2$ value. This sampling and evaluation process is repeated 10 times, with the resulting metrics being averaged and presented in Table 2.

It is apparent from the results that ThermoLearn significantly outperforms the other algorithms, exhibiting even greater (70% for JANAF and 60% for PhononDB) margins of improvement compared to those observed in Table 1. The incorporation of thermodynamic constraints in ThermoLearn enables it to acquire domain-specific knowledge, which significantly enhances its ability to generalize across data that is inherently different from that used in training.

### Physical insights

Constraining the architecture to follow the law of thermodynamics results in a configuration where the penultimate layer is engineered to produce values corresponding to Energy and Entropy. Subsequently, the final layer with fixed weights and zero bias produces Free Energy.

We test our model for the metal oxide PhononDB dataset which has just 873 entries. We use 4-fold cross-validation to compute the performance of the model on the full dataset. The penultimate and final layer outputs vs their respective ground truth values are shown in Fig. 4. This shows the efficacy of ThermoLearn across three distinct outputs. Notably, an exceptional coefficient of determination ($R^2$) of 0.99 has been attained for all three outputs. This achievement highlights how well the model can accurately capture complex patterns within the dataset. However, it should be noted that since the target quantity for our study is Gibbs free energy, we use higher penalties on free energy deviations during training. Conversely, should the target variables be energy or entropy, a similar strategy can be applied for optimal results. Moreover for the JANAF dataset, the ThermoLearn does equally well for all three thermodynamic quantities. This has been showcased in the Fig. S1.

**Fig. 5:**
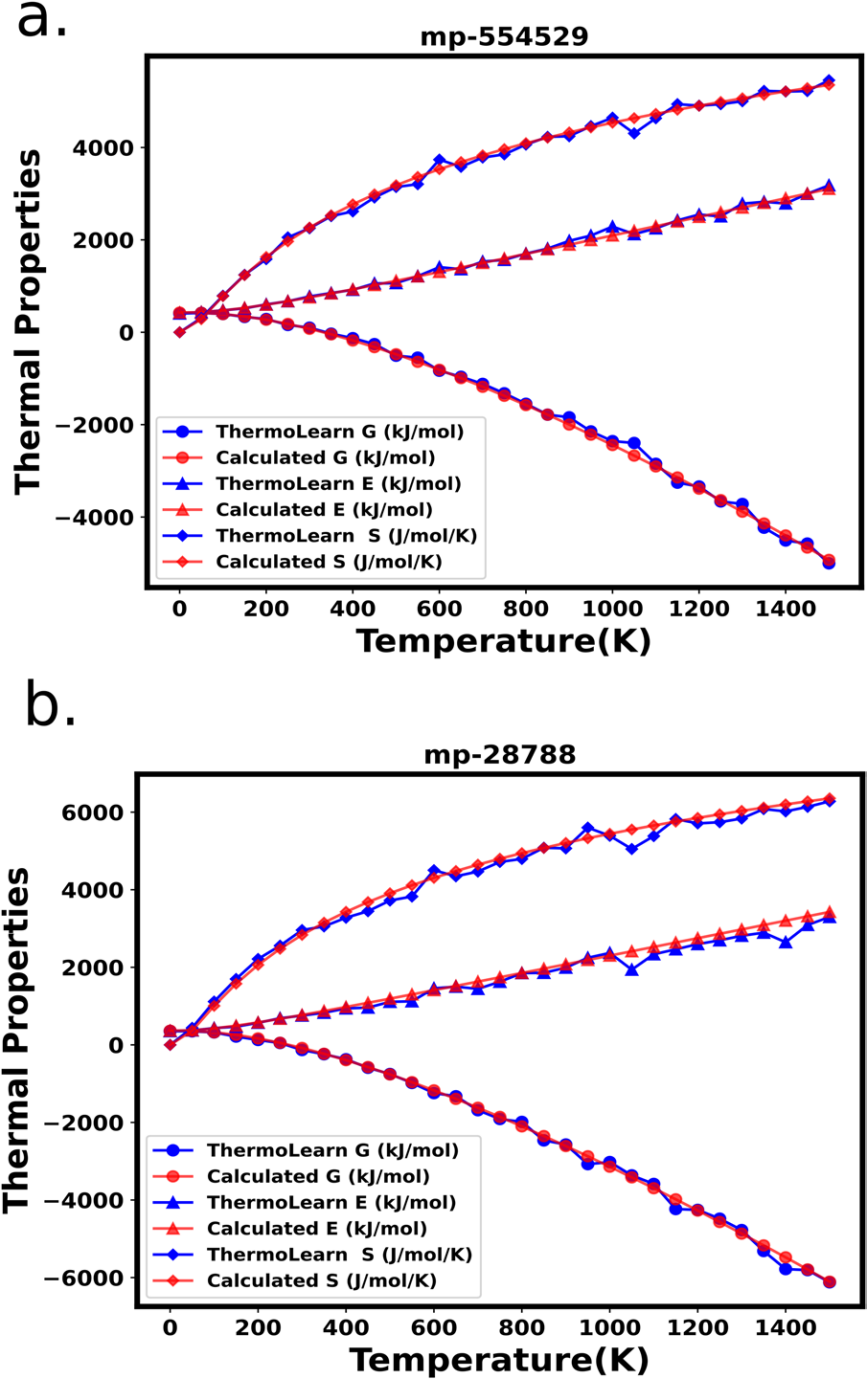
Comparison of predicted (thermoLearn) versus calculated thermodynamic quantities as a function of temperature for **a** mp-554529[$\textrm{Ba}_2\textrm{TiO}_4$], **b** mp-28788[$\textrm{Ba}_9(\textrm{AuO}_6)_2$]

Till now we have benchmarked the performance of ThermoLearn across various materials over a fixed temperature. To further evaluate the efficacy of ThermoLearn, we conducted a comprehensive analysis across a spectrum of temperatures using the PhononDB dataset. Multiple models of ThermoLearn are trained on temperatures ranging from 50 to 1500 K in increments of 50 K. Subsequently, the performance of these instances is assessed through rigorous testing on unseen materials. Figure 5 illustrates two such test cases, showcasing the performance of ThermoLearn for all three thermodynamic quantities across various temperatures. This plot verifies the robustness of the model across various temperatures.

In the featurization section, it is noted that either crystal composition features or CGCNN features may be employed to construct the ML model utilizing the phononDb dataset. As depicted in SI (Table in SI), ThermoLearn demonstrates superior performance compared to the conventional neural network, regardless of the chosen featurization scheme. This indicates that advanced featurization techniques, as utilized by algorithms such as CGCNN and M3GNET, can be adopted. By fine-tuning the final layers of the network to incorporate physical insights derived from thermodynamic equations, the prediction of free energy is enhanced. This adaptability of ThermoLearn opens up potential applications to vastly different systems, such as proteins and biomolecules.

## Conclusion

In summary, the development of ThermoLearn represents a significant advancement in predictive modeling for thermodynamic properties. Its unique multi-output architecture allows for the simultaneous prediction of key parameters such as Gibbs free energy, total energy, and entropy, providing a comprehensive understanding of material behavior. ThermoLearn outshines existing algorithms in terms of accuracy and robustness across multiple datasets, including experimental and computational ones. Moreover, the architecture of ThermoLearn is distinctively designed to integrate domain-specific knowledge, substantially improving its efficacy on datasets not encountered during training. Its robustness is consistently demonstrated through its stable performance across diverse feature representation techniques, including crystal-compositional and graph-based features. This versatility originates from ThermoLearn’s profound grasp of fundamental physical principles, enabling it to deliver precise predictions across a range of methodologies. The model is very general and can be utilized for various biochemical and material datasets. Furthermore, we believe that ThermoLearn can be extended and generalized to incorporate additional thermodynamic equations, such as the Eyring equation for rate constants and Gibbs free energy. Overall, ThermoLearn presents a promising approach for accurately predicting thermodynamic properties in chemistry for small datasets, offering potential applications in various research domains.

## Supplementary Information

Supplementary material 1.

## Data Availability

All the codes and data are available in https://github.com/Sudo-Raheel/ThermoLearn.
