# Supplementary material for: Advancements in thermochemical predictions: a multi-output thermodynamics-informed neural network approach
Source: J Cheminform. 2025 Jun 16;17:95. doi: 10.1186/s13321-025-01033-0 (PMC12168259; doi:10.1186/s13321-025-01033-0)
Supplement: Supplementary file 1 — Supplementary material 1. [file 13321_2025_1033_MOESM1_ESM.pdf]

Enhancing Thermochemical Predictive Accuracy with Thermodynamics-Informed Multi-Output Neural Networks

Raheel Hammad* and Sownyak Mondal*
Tata Institute of Fundamental Research Hyderabad, Hyderabad-500046, Telangana, India.
E-mail: raheelhammad@tifrh.res.in; msownyak@tifrh.res.in

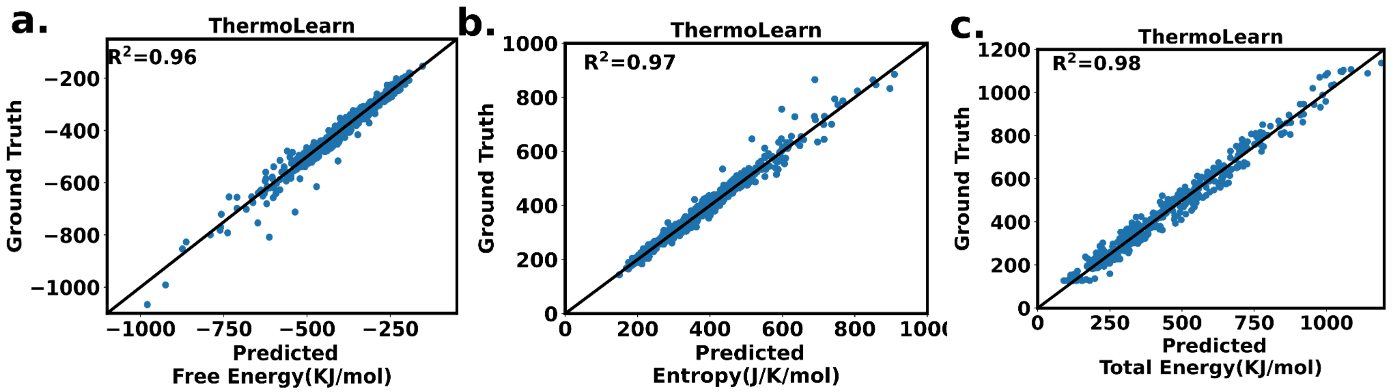

FigureS1: Predicted values vs the Ground truth values of Free Energy, Entropy and Total energy respectively for the NIST-JANAF dataset.

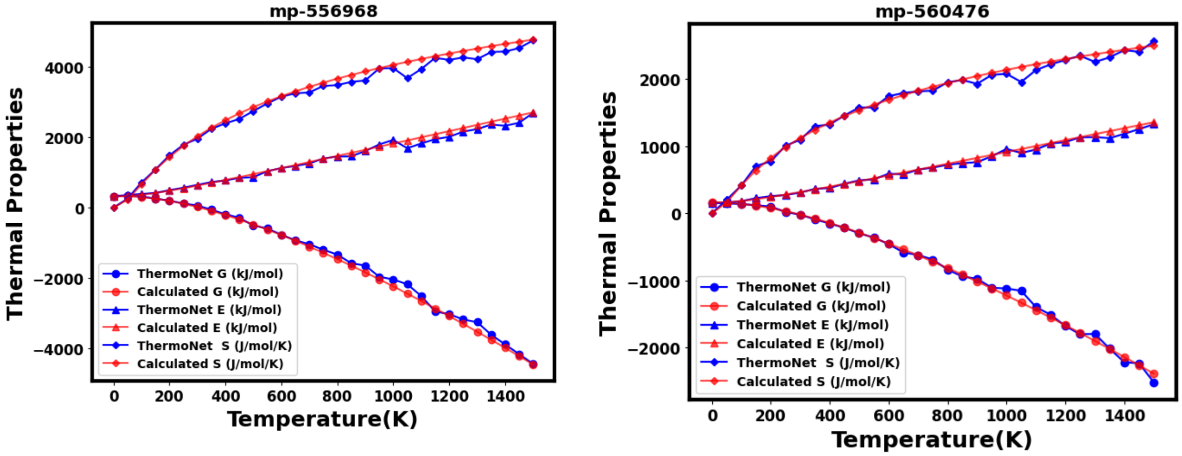

FigureS2: Comparision of Predicted(ThermoLearn) vs Calculated Thermodynamic quantities as a function of temperature for a)$RbNa_3SnO_4$ b)Rb_3NaTiO_4

TableS1: ThermoLearn vs Vanilla Neural Network performance for PhononDB dataset with graph based (unsupervised) features.

Methods/Metrics	R^2	RMSE(kJ/mol)	Feautrization
NN	0.33	106.35	CGCNN(unsupervised)
**ThermoLean**	**0.38**	**101.8**	CGCNN(unsupervised)
NN	0.94	31.9	CGCNN(supervised)
**ThermoLean**	**0.95**	**29.84**	CGCNN(supervised)

Unsupervised /Supervised featurization using CGCNN has been shown in https://github.com/Sudo-Raheel/ThermoLearn.

Thermolearn Architecture and Training Parameters for NIST-JANAF and PhononDB Datasets

Description	NIST-JANAF Dataset	PhononDB Dataset
**Temperature**	1200	300
**Input Features (input_dim)**	25	30
**Hidden Layers**	3 fully connected layers	3 fully connected layers
**Layer 1**	Fully Connected (25 → 12 neurons) + Leaky ReLU	Fully Connected (30 → 15 neurons) + Leaky ReLU
**Layer 2**	Fully Connected (12 → 6 neurons) + Leaky ReLU	Fully Connected (15 → 7 neurons) + Leaky ReLU
**Layer 3**	Fully Connected (6 → 2 neurons) + Leaky ReLU	Fully Connected (7 → 2 neurons) + Leaky ReLU
**Output Layer**	Fully Connected (2 → 1 neuron) + Leaky ReLU	Fully Connected (2 → 1 neuron) + Leaky ReLU
**Activation Function**	Leaky ReLU	Leaky ReLU
**Loss Function**	Mean Squared Error (MSE)	Mean Squared Error (MSE)
**Learning Rate (lr)**	0.1	0.01
**Batch Size**	32	32
**Number of Epochs**	300	300
**Optimizer**	Adam	Adam
**Scaling Method**	MinMaxScaler	MinMaxScaler
**w1**	4	9
**w2**	3	3.3
**w3**	4	110

Used features of both NIST-JANAF and PhononDB dataset:

NIST-JANAF Features	PhononDB Features
nsites	nsites
avg_Period	density_atomic
avg_crystal radius	avg_crystal radius
avg_Atomic_Weight	volume
avg_specific heat (J/g K)	mean_bond
avg_Density (g/mL)	outer_density
avg_Atomic_Number	avg_Period
min_crystal radius	reduced_electron_density
avg_Covalent_Radius	energy_per_atom
avg_ionic radius	formation_energy_per_atom
avg_electron affinity (kJ/mol)	min_bond
avg_Atomic Radius	C_lat
min_specific heat (J/g K)	avg_ionic radius
avg_valence d	avg_Covalent_Radius
avg_number of valence electrons	max_bond
diff_Mendeleev Number	avg_Atomic Radius
max_valence d	min_crystal radius
diff_electron affinity (kJ/mol)	avg_Mullinke EN
max_Atomic_Number	avg_Zunger radii sum
avg_1st ionization potential (kJ/mol)	avg_specific heat (J/g K)
min_Atomic_Weight	avg_valence d
avg_heat atomization (kJ/mol)	avg_1st ionization potential (kJ/mol)
max_Zunger radii sum	B_lat
avg_Zunger radii sum	band_gap

min_Atomic_Number	avg_Atomic_Weight
	reduced_gilman_density
	reduced_valence_density
	max_1st ionization potential (kJ/mol)
	avg_Gordy electronegativity
	avg_heat of vaporization (kJ/mol)
